# Vaccination with Replication Deficient Adenovectors Encoding YF-17D Antigens Induces Long-Lasting Protection from Severe Yellow Fever Virus Infection in Mice

**DOI:** 10.1371/journal.pntd.0004464

**Published:** 2016-02-17

**Authors:** Maria R. Bassi, Mads A. B. Larsen, Michael Kongsgaard, Michael Rasmussen, Søren Buus, Anette Stryhn, Allan R. Thomsen, Jan P. Christensen

**Affiliations:** Department of Immunology and Microbiology, University of Copenhagen, Copenhagen, Denmark; Florida Gulf Coast University, UNITED STATES

## Abstract

The live attenuated yellow fever vaccine (YF-17D) has been successfully used for more than 70 years. It is generally considered a safe vaccine, however, recent reports of serious adverse events following vaccination have raised concerns and led to suggestions that even safer YF vaccines should be developed. Replication deficient adenoviruses (Ad) have been widely evaluated as recombinant vectors, particularly in the context of prophylactic vaccination against viral infections in which induction of CD8^+^ T-cell mediated immunity is crucial, but potent antibody responses may also be elicited using these vectors. In this study, we present two adenobased vectors targeting non-structural and structural YF antigens and characterize their immunological properties. We report that a single immunization with an Ad-vector encoding the non-structural protein 3 from YF-17D could elicit a strong CD8^+^ T-cell response, which afforded a high degree of protection from subsequent intracranial challenge of vaccinated mice. However, full protection was only observed using a vector encoding the structural proteins from YF-17D. This vector elicited virus-specific CD8^+^ T cells as well as neutralizing antibodies, and both components were shown to be important for protection thus mimicking the situation recently uncovered in YF-17D vaccinated mice. Considering that Ad-vectors are very safe, easy to produce and highly immunogenic in humans, our data indicate that a replication deficient adenovector-based YF vaccine may represent a safe and efficient alternative to the classical live attenuated YF vaccine and should be further tested.

## Author Summary

Live attenuated yellow fever vaccine (YF-17D) is an efficient and generally safe vaccine. Nevertheless, in recent years the reporting of serious adverse effects together with the given limitations in the use of this live vaccine in certain risk groups has spurred an interest in developing a more generally applicable and safer alternative. Using an adenovector platform and recombinant vaccines targeting both structural and non-structural YF antigens, we now demonstrate that non-replicating adenobased vaccines may be used to induce a state of host immunity, which like YF-17D vaccination encompasses both major arms of the adaptive immune system. Furthermore, in a murine challenge model, adenovector induced protection fully matched that induced by the current vaccine. Taken together our results strongly suggest that adenovectored vaccines targeting structural and non-structural viral antigens represent a viable and safe alternative to the existing live, attenuated YF vaccine.

## Introduction

The design of vaccines against viral infections has evolved considerably with the advances in molecular biology, which have created many alternative approaches to the empirical development of live vaccines. Thus, the first generation of live attenuated vaccines and the second generation of subunit vaccines have now been followed by a third generation of vaccines based on recombinant DNA technology. The newly designed vaccines have several advantages compared to empiric attenuated live vaccines: their production is faster, cheaper and easier to control, and, importantly, their safety profile is considerably better than that of live viruses making them more appealing for use in humans. However, they have rarely shown the same immunogenicity as their live predecessors, and the biological mechanisms behind this difference have been the subject of extensive research.

The yellow fever (YF) vaccine, based on the live attenuated YF-17D virus, was developed in the 1930s by serial tissue culture passage of wild type YF virus (YFV) in mouse and chicken cell cultures [1–3]. A single vaccination with YF-17D can confer protection in more than 95% of the vaccinees, and immunity has been shown to last up to 40 years post vaccination and to correlate with presence of neutralizing Abs [4,5]. In spite of the clear success in preventing infection with YFV in many areas of the world, the YF-17D vaccine also has its dark side; rare, but often fatal vaccine-associated adverse events (SAEs) may be induced [5]. These SAEs mainly fall into two categories: vaccine-associated neurotropic disease (YEL-AND), which consists in a post-vaccinal encephalitis [5,6], and vaccine-associated viscerotropic disease (YEL-AVD), which is a pansystemic infection characterized by liver damage, similarly to infection with wild type YFV [7–9]. Interestingly, sequence analysis of the few isolates obtained from patients in whom adverse events following vaccination were fatal, demonstrated that the virus had not reverted to virulence, rather host genetic factors appeared to be responsible for the severe reaction to YF-17D virus [5,10]. Moreover, due to its live viral nature, the YF vaccine is contraindicated in pregnant women, infants, elderly, immunosuppressed and certain HIV infected individuals as well as in people with hypersensitivity to eggs in which the vaccine is still manufactured [5]. In this perspective, implementation of alternative vaccine strategies such as DNA-based vaccines has become desirable.

Recombinant DNA vaccines in which the antigen is encoded by an attenuated viral vector have demonstrated great potential, and very recently it has been found that a DNA vaccine encoding the envelope antigen of YFV may induce protection in murine studies [11]. However, the immunogenecity of naked DNA vaccines is substantially surpassed by that of replication deficient adenoviral vectors, which have been found to represent very efficient immunogens also in primates [12,13]. Thus, vaccination with recombinant adenovectors has been shown to elicit strong cellular immunity against antigens from several different viral pathogens such as Ebola virus, lymphocytic choriomeningitis virus, HIV, hepatitis C virus [14–19], as well as non-viral pathogens such as Listeria monocytogenes [20] and malaria [21]. In relation to YFV infection, recent results from our group strongly suggest that long-term clinical protection is mediated not only by neutralizing antibodies as previously believed [4], but also by virus-specific CD8^+^ T cells [22]. Therefore, with the dual purpose of using adenoviral vectors for further studies of immunity to YFV infection in our intracranial challenge mouse model and to explore the possibility of developing an adenobased vaccine alternative, we generated two vector constructs: an Ad serotype 5 (Ad5) vaccine vector encoding the YF-17D structural proteins core (C), membrane (M) and envelope (E), as well as an Ad5 vector encoding the viral non-structural protein 3 (NS3). The rationale behind this strategy was that the former vector would likely induce a humoral response towards the encoded YF-17D surface antigens, while the latter vector would induce cell-mediated immunity toward antigens present within YFV infected cells. Our results showed that vaccination with Ad-YF C,M,E or Ad-YF NS3 both resulted in long-standing protection of mice from severe YFV infection. While virus-specific CD8^+^ T cells appear to represent the major effectors when the viral NS3 protein was chosen as antigen, a combination of T-cell and B-cell immunity determined the outcome in mice vaccinated with the vector encoding the three viral structural proteins. Thus, besides strengthening the evidence for a role of CD8^+^ T cells in protection against severe YFV infection, our findings point to adenoviral vectors as potential vaccine candidates that may be used to develop a safer alternative for immunization against YFV infection.

## Materials and Methods

### Mice

Female C57BL/6 (wt B6) mice, B-cell (μMT/μMT) deficient mice, β_2_-microglobulin (β_2_m^-/-^) and MHC class II (A_β_^-/-^) deficient mice, as well as mice deficient in H-2K^b^ or in both H-2K^b^/H-2D^b^ molecules, were all obtained from Taconic Farms. Perforin (Prf) deficient and IFN-γ deficient mice were originally obtained from The Jackson Laboratory and IFN-γ/perforin double-deficient (IFN-γ/Prf) mice were generated locally through intercrossing of these strains, as previously described [23]. All mice used in this study were 7–10 weeks old and housed in a pathogen–free facility. All experiments were approved by the national animal ethics committee and performed in accordance to the national guidelines.

### Ethics statement

Experiments were conducted in accordance with national Danish guidelines (Amendment # 1306 of November 23, 2007) regarding animal experiments as approved by the Danish Animal Experiments Inspectorate, Ministry of Justice, permission number 2009/561-1679. The mice were housed in accordance with good animal practice as defined by FELASA.

### Recombinant adenoviral vectors

pacCMV shuttle vectors encoding the structural proteins core, membrane and envelope (C, M, E), and the nonstructural protein 3 (NS3) from YF-17D virus (GenBank: X03700.1) were obtained from GenScript (Piscataway, NJ, USA). pacCMV vectors encoding the truncated form of NS3 (aa. 201–325) were also obtained from Gen Script (Piscataway, NJ, USA).

**Fig 1:**
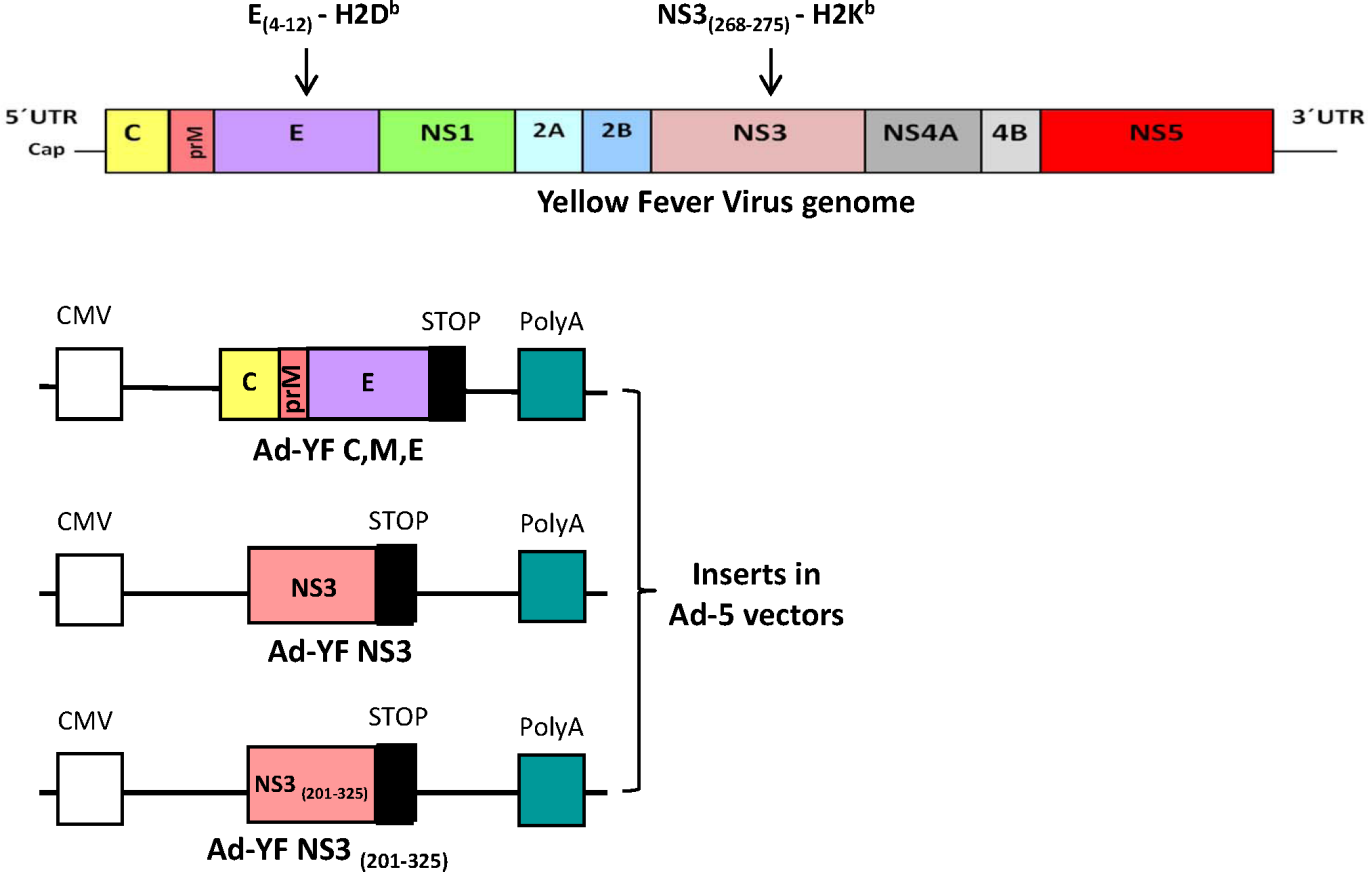
Schematic representation of the YFV virus genome and of the recombinant adenoviral vectored-vaccines used in this study. Three vectors encoding antigens from the YF-17D virus were used: Ad-YF C,M,E, Ad-YF NS3 and Ad-YF NS3_(201–325_). The positions of the two previously identified murine MHC class I-restricted viral epitopes have been indicated.

From all shuttle plasmids, human type 5 recombinant adenovirus (Ad5) vectors were produced through homologous recombination by standard methods [24]. After purification on a cesium chloride (CsCl) density gradient, adenoviral stocks were immediately frozen as single use aliquots in 10% glycerol at -80°C, and the infectivity of the stocks was determined using the Adeno-X Rapid Titer kit (BD Clontech). The presence of the inserted transgene was validated in all the recombinant Ad5 vectors by sequence analysis prior to their use for vaccination in mice. The genome of YF-17D virus with the position of the two known class I-restricted epitopes in B6 (H-2^b^) mice and the derived Ad-YF vectors, are depicted in Fig 1.

### YF-17D virus preparation and quantitation

YF-17D virus (Stamaril, Sanofi Pasteur; reconstituted as recommended by the manufacturer) was propagated in Vero (ATCC CCL-81) cells grown in DMEM containing 10% FCS, glutamine and antibiotics (penicillin and streptomycin). Cell monolayers were seeded 24 hours earlier and infected with at a multiplicity of infection (MOI) of 0.001/cell in DMEM 2% FCS; infectious supernatants were harvested when cytopathic effect was more than 60% (day 6 p.i), freeze-thawed and clarified of cell debris at 2000 rpm for 15 min at 4C°. Viral stocks were stored as single use aliquots at -80C°. For determining virus infectious titers, we used an Immuno Focus assay (IFA) recently developed in our lab [22]. In brief, virus stocks were serially diluted (ten-fold) in DMEM and adsorbed for 1h at 37 C° onto Vero (ATCC CCL-81) cell monolayers in twenty four-well plates; cells were then overlaid with media containing 0.9% methyl cellulose and incubated at 37 C° for 3 days. Following fixation and permeabilization, a monoclonal antibody directed against NS3 from YF-17D virus was used for detection of virus antigens within infected cells; the foci of infection (analogous to plaques) were visualized using HRP-substrate reaction and counted. When virus titers in the brains were determined, the IFA was performed on serial 10-fold dilutions of homogenized and clarified 10% organ suspensions.

### Plaque reduction neutralization test

Neutralizing antibodies to YF-17D virus in sera from vaccinated mice were measured as described previously [22]. Briefly, appropriately diluted YF-17D virus (resulting in about 70–100 pfu per well) was incubated for 1 hour at 37°C with serial (2-fold) dilutions of mouse sera obtained from individual mice vaccinated with Ad-YF C,M,E or Ad-YF NS3. The virus-serum mixture was subsequently added to Vero cells monolayers in 24-well plates and incubated for 2 hours at 37°C, after which the overlay media containing 0.9% methyl cellulose was added. After 3 days of incubation at 37°C, the overlay media was removed and, following fixation and permeabilization, cells were stained as described above (IFA). Following counting, the neutralizing antibody titer, defined as the highest serum dilution neutralizing more than 50% of the viral plaques, was determined.

### Vaccination and challenge

For adenovirus vaccination, 2x10^7^ pfu of recombinant Ad-5 in a volume of 30 μl was given subcutaneously to isofluorane-anesthetized mice in the right hind foot pad; vaccination with YF-17D virus at the dose of 10^5^ pfu in 300 μl was given subcutaneously (s.c.) at the base of the tail.

In YFV challenge studies, deeply anesthetized mice were injected intracranial with 10^4^ pfu of YF-17D virus in a volume of 30 μl; when assessing clinical protection, mice were checked twice daily for two weeks following intracranial challenge and euthanized when development of illness (drop in body weight, ruffled fur, twitching) together with neurologic signs (hind leg paralysis and weakness) were observed. Mice developing sickness within 4 days post challenge were excluded from experiments.

### In vivo depletion of T cells

A combination of two monoclonal antibodies (YTS 169 and YTS 156)[25,26] was used for in vivo depletion of CD8^+^ T cells from vaccinated mice prior to challenge. Mice to be depleted were injected i.p. with 100 μg of antibody one day prior to i.c challenge and at days 1 and 4 post challenge. The efficiency of the depletion was confirmed by flow cytometric analysis at day 7 post challenge.

### Spleen cell preparation and flow cytometry

Single cell suspensions of splenocytes were obtained by pressing the organs through a fine steel mesh (mesh size, 70 μm), followed by centrifugation and re-suspension in RPMI 1640 cell culture medium. The frequencies of antigen-specific CD8^+^ T cells in the spleen were determined by intracellular cytokine staining (ICS) performed after 5 h of incubation with relevant peptides (0.1 μg/ml of NS3 _(268–275)_ or E _(4–12)_) in the presence of monensin (3 μM) at 37°C in 5% CO2. After incubation, the cells were stained with Abs for cell surface markers (peridinin chlorophyll protein-Cy5.5- or BrilliantViolet 421-CD8, APC-Cy7-CD44) and Ab for intracellular cytokine (APC- interferon gamma [IFN-γ]). Samples were run on an LSRII flow cytometer (BD biosciences) and analyzed using FlowJo software (TreeStar).

### Statistical analysis

A nonparametric Mann-Whitney U test was used to compare quantitative data; survival after viral challenge was analyzed using the Log Rank test (*p < 0.05; **p < 0.01; (***p < 0.001). GraphPad Prism (version 6) software was used for statistical analysis.

## Results

### Ad-YF vectors targeting both structural and non-structural gene products induce relevant antiviral immunity

**Fig 2:**
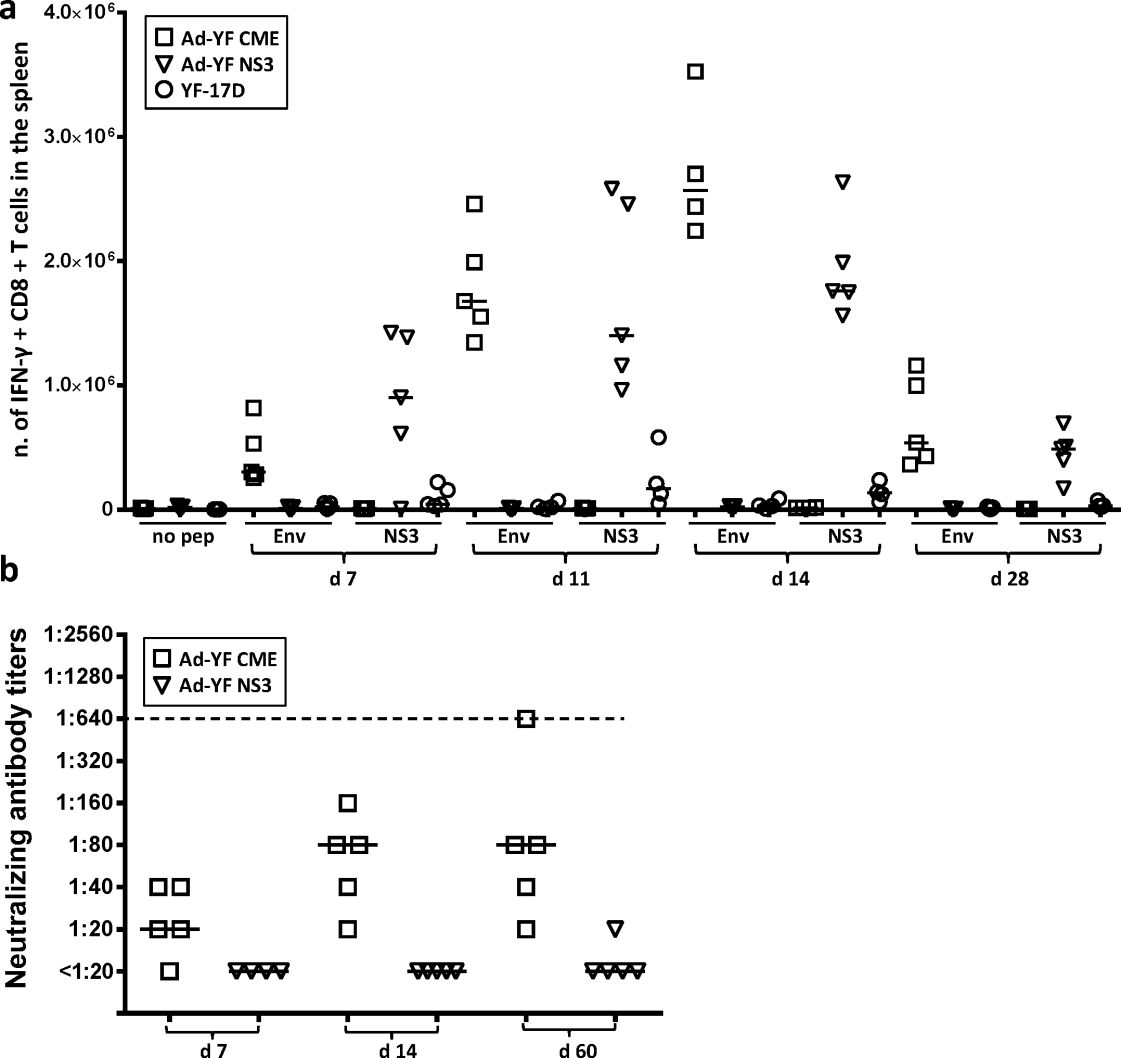
Analysis of immune responses induced by vaccination with Ad-YF vectors. WT B6 mice were vaccinated with Ad-YF C,M,E, Ad-YF NS3 or YF-17D virus. **a)** Numbers of CD44^+^IFN-γ^+^ CD8^+^ T cells following ex vivo peptide stimulation with NS3_(268–275)_ and E_(4–12)_ are depicted as a function of time after vaccination; non-stimulated cells serve as controls; symbols denote individual animals. **b)** Neutralizing antibody titers as a function of time after vaccination. The dashed line indicates the median titer of the neutralizing antibody measured in WT B6 mice vaccinated with YF-17D virus (cf. ref. (21)); symbols denote individual animals.

As a first approach to evaluate our YF vaccine candidates, we analysed the immunogenicity of our structurally and non-structurally targeted vectors (see Fig 1) in terms of their capacity to induce effector CD8^+^ T cells and virus-specific antibodies in vaccinated mice. From previous studies it is known that both vectors contained T-cell epitopes of relevance in H-2^b^ mice: a dominant H-2K^b^-restricted epitope has been mapped to the NS3 protein, and an H-2D^b^-restricted subdominant epitope can be found in the viral E protein [27]. Consequently, to evaluate the CD8^+^ T-cell responses elicited in vaccinated mice, wt B6 mice were inoculated in the f.p. with 2x10^7^ pfu of either of the two prototypic Ad5 vaccines, and the kinetics of the primary CD8^+^ T-cell responses was followed by flow cytometric analysis of ICS; a group of mice vaccinated with 10^5^ pfu of YF-17D virus s.c. was included for comparison. As can be seen in Fig 2A, both constructs induced strong CD8^+^ T-cell responses against the YFV epitope encoded by the vector in question. Furthermore, it should be noted that the magnitude of T-cell responses generated in adenovector vaccinated mice markedly surpassed those elicited by the YFV vaccine; whether this is also the case in humans we do not know, but certainly adenobased vaccines are highly immunogenic also in primates [12,13].

Besides CD8+ T-cell responses, we also measured the antibody responses induced in vaccinated mice by an in vitro neutralization assay. As can be seen in Fig 2B, the Ad-YF C,M,E vector expressing the surface antigens of the YFV induced a clear virus-neutralizing response, albeit not quite at the level found subsequent to YF vaccination [22]. In contrast, mice vaccinated with the vector encoding NS3 did not generate any antibodies detectable in the neutralization assay. From these results we conclude that Ad-YF C,M,E induces both a humoral and a cell-mediated immune response of potential relevance to in vivo protection, while Ad-YF NS3 induces only a relevant cell-mediated response.

### Vaccination with both Ad-YF vectors confers protection against YF intracranial challenge

**Fig 3:**
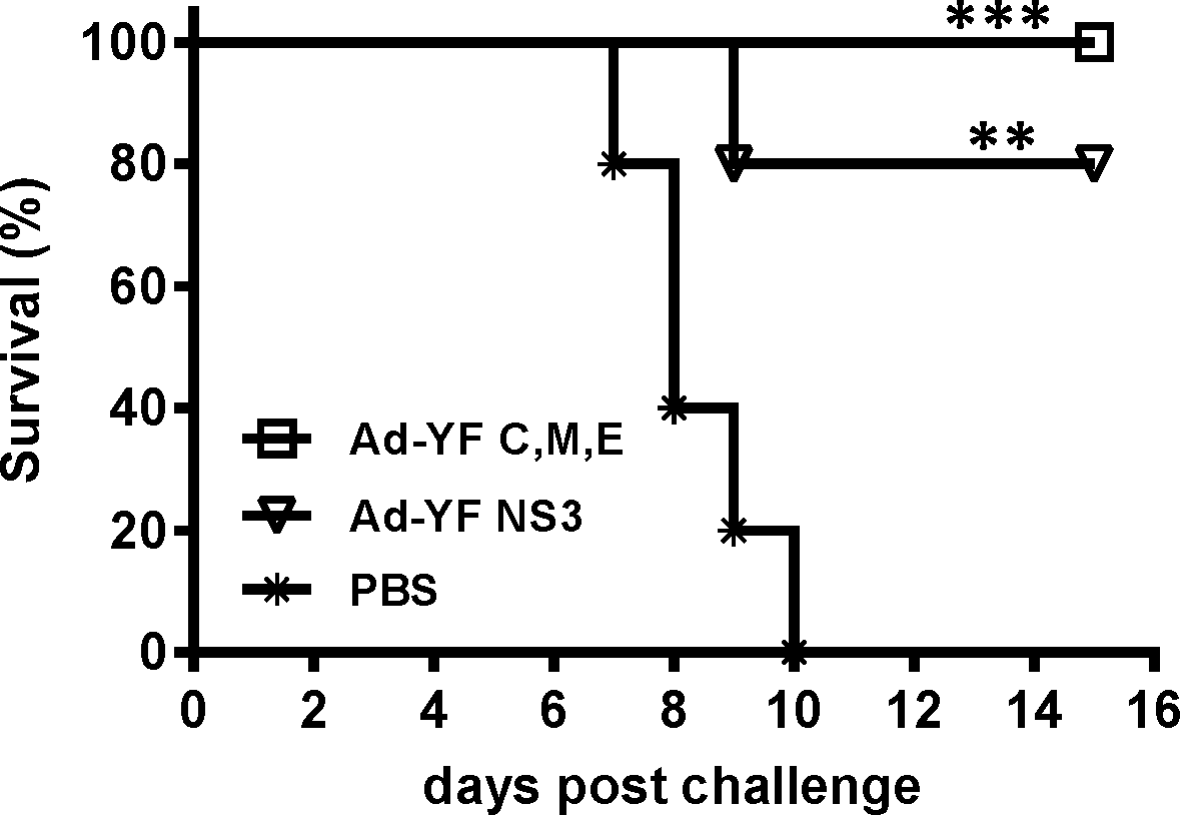
In vivo protection induced by vaccination with Ad-YF vectors. WT B6 mice were vaccinated with Ad-YF C,M,E or Ad-YF NS3 and intracranial challenged with YF-17D virus. Survival of vaccinated mice are compared to that of sham vaccinated (PBS) animals. N = 5 in all groups. Experiment performed twice with similar results.

Having determined the type and magnitude of immune responses elicited by each of our adenoviral vectors, we proceeded to test the protective efficacy of a single immunization with either of the two Ad-YF vectors. Mice were inoculated with 2x10^7^ pfu of Ad-YF C,M,E, Ad-YF NS3 or PBS in the f.p., and 14 days later, all animals were intracranial challenged with 10^4^ pfu of YF-17D virus (Fig 3). The intracranial challenge model we adopted has previously been shown to induce a uniformly fatal neurotropic disease in naïve B6 mice, while YFV vaccinated mice are protected from disease [22]. We found that vaccination with Ad-YF C,M,E resulted in 100% protection from viral challenge, while all sham-vaccinated (PBS) mice succumbed to the infection (Fig 2). Interestingly, vaccination with Ad-YF NS3 resulted in 80% protection from subsequent YF challenge. Since this vector only encodes a non-structural viral protein, which is found exclusively in infected host cells, and since we have previously demonstrated that NS3 specific CD8^+^ T cells infiltrate the YF-infected brain [22], the latter observation strongly supported the idea that protection in this animal model of YFV infection may be accomplished by T cells alone.

### Protection afforded by Ad-YF NS3 vaccination relies on CD8^+^ T-cell responses

To confirm the hypothesis that T-cell immunity suffices for protection, we first evaluated the outcome of YFV challenge in several mouse knock-out (KO) strains carrying targeted deficiencies in CD8^+^ T-cell responses or in their effector molecules. Thus, wt B6 mice, β_2_m KO mice, H-2k^b^ KO mice and IFN-γ/Prf double KO mice were vaccinated with Ad-YF NS3; 14 days later, the mice were intracranial challenged with 10^4^ pfu of YF-17D virus, and the clinical outcome was monitored. Additionally, MHC class II KO mice were included in the experiment to examine the role of CD4^+^ T cells in the Ad-YF NS3 induced response.

**Fig 4:**
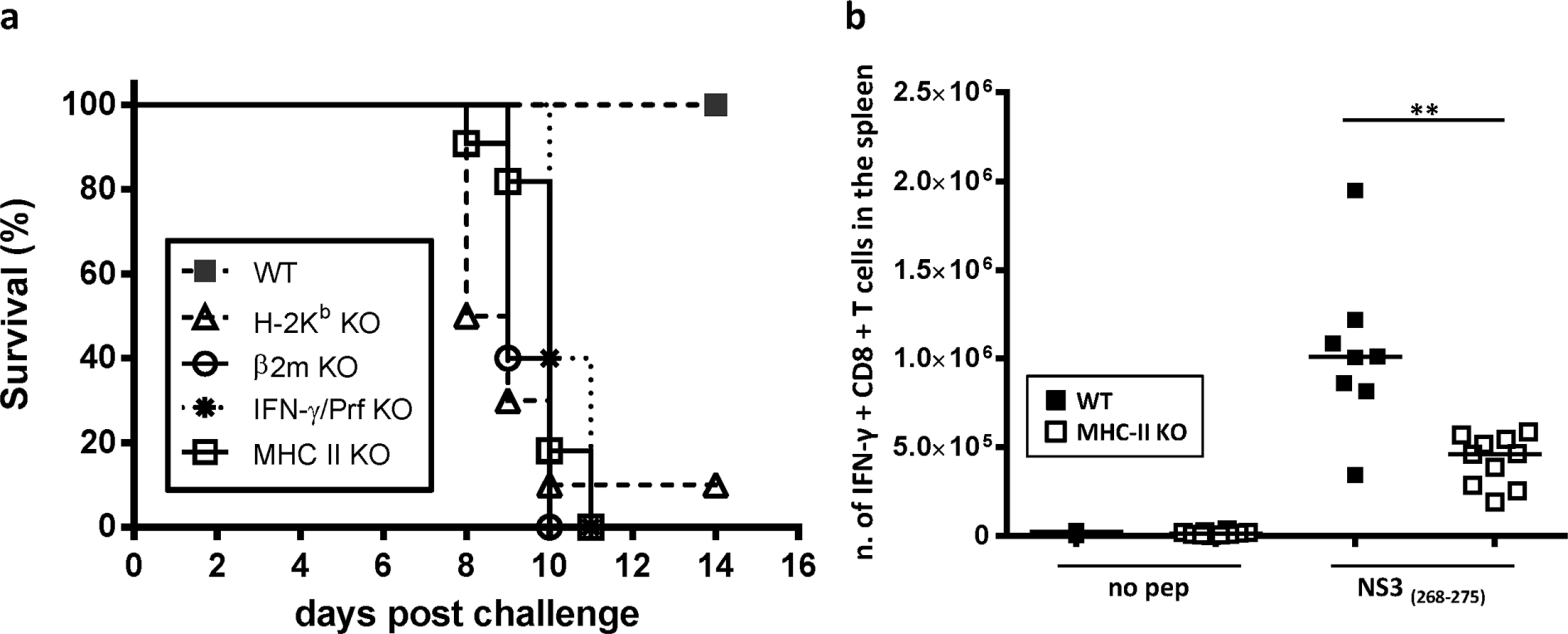
Role of CD8^+^ T cells in mediating protection from YF viral challenge following Ad-YF NS3 vaccination. WT B6 mice, H-2K^b^ KO mice, β2m KO mice, IFN-γ/Prf double KO mice and MHC class II KO mice were vaccinated with Ad-YF NS3 and 14 days later intracranial challenged with YF-17D virus. **a)** Survival curves are depicted; n = 5 in WT group and 10 in all other groups. **b)** Numbers of NS3_(268–275)_—specific CD8^+^ T cells in the spleen of WT B6 mice and MHC class II KO mice 8 days post Ad-YF NS3 vaccination are enumerated. Results are pooled from two independent experiments; symbols denote individual animals.

As can be seen in Fig 4A, we found that NS3 induced protection was completely abrogated in β_2_m KO mice (no survival), and profoundly impaired in H-2K^b^ KO mice, in which case only 10% of vaccinated animals survived the challenge, whereas the survival of wt B6 mice in this experiment was 100%. Given that the H-2K^b^ molecule is the restriction element for the dominant NS3_(268–75)_ epitope [27], this result strongly support the idea that the CD8^+^ T cells specific for this epitope may be accountable for the vector-induced immunity; however, formally we cannot rule out the contribution of additional H-2K^b^-restricted epitopes potentially present in the NS3 protein. Consistent with a dominant role for effector T cells, the combined deficiency of IFN-γ and perforin also resulted in complete abrogation of protection, with no survivors recorded among vaccinated IFN-γ/Prf double KO mice (Fig 4A). Thus, we concluded that the Ad-YF NS3 vector induces immunity to YFV via IFN-γ and/or perforin producing CD8^+^ T cells specific for viral H-2k^b^-restricted epitopes.

Notably, we also found that the absence of CD4^+^ T cells resulted in a total suppression of protective immunity (no survival in MHC class II KO mice) (Fig 4A). This cannot reflect lack of help to B cells, since the NS3 vaccine does not induce protective antibodies. However, a requirement for CD4^+^ T cell help for an effective CD8^+^ T cell response to Ad5-encoded viral antigens has previously been demonstrated [28]. Therefore, to test whether this was also the case for NS3 from the YF-17D virus, MHC class II KO mice, as well as wt B6 mice, were vaccinated with Ad-YF NS3, and the splenic CD8^+^ T cell response was analyzed by ICS at day 8 post vaccination. We found that MHC class II KO mice presented a significantly reduced NS3 _(268–75)_ specific CD8^+^ T-cell response as evident by markedly lower numbers of IFN-γ producing CD8^+^ splenocytes in these mice compared to wt B6 animals (Fig 4B). Taken together, these findings imply that CD8^+^ T cells are the major effectors in the immune response induced by Ad-YF NS3 vaccination, and that CD4^+^ T cell help is essential in the priming phase. Whether CD4^+^ T cells are also important at later stages of the immune response cannot be established from our data, but “helpless” CD8^+^ T cells are known to be inferior with regard to survival and ability to undergo secondary expansion [29,30].

### Viral loads in the brains of mice vaccinated with Ad-YF NS3 vectors match CD8^+^ T-cell activity

**Fig 5:**
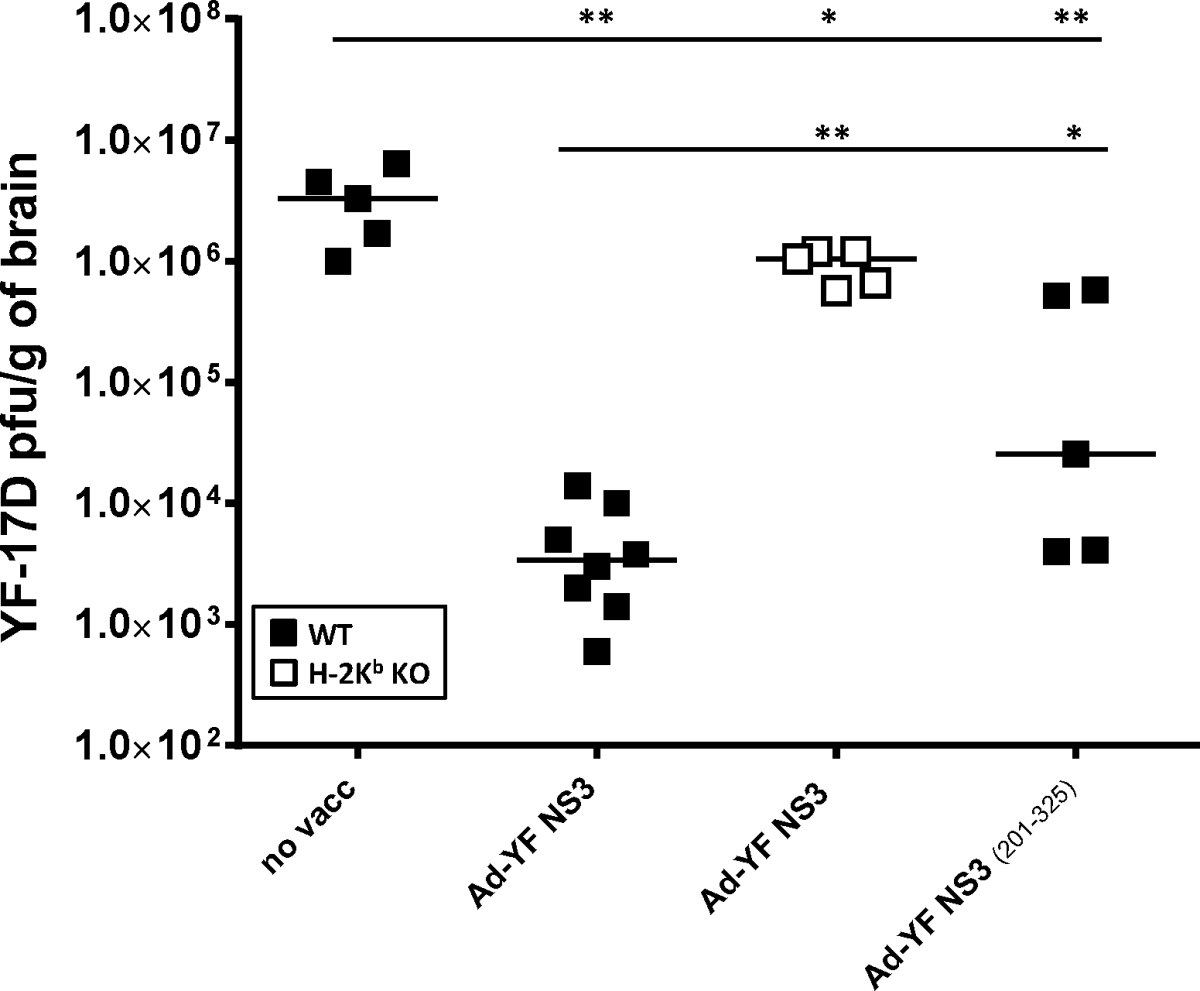
Viral loads in the brains of mice vaccinated with Ad-YF NS3 or Ad-YF NS3 _(201–325)_ reflect CD8^+^ T cell numbers. Non-vaccinated, Ad-YF NS3 vaccinated or Ad-YF NS3 _(201–325)_ vaccinated WT B6 mice (filled squares) as well as Ad-YF NS3 vaccinated H-2K^b^ KO mice (open squares) were challenged with YFV intracranial 14 after vaccination, and viral titers in the CNS were determined 7 days later. Symbols denote individual animals.

The fact that Ad-YF NS3 induced protection depends on cell-mediated immunity, suggested to us that, upon intracranial challenge, the virus inoculum would have to be cleared from the CNS of wt B6 mice by CD8^+^ effector T cells. As we had previously observed that lack of H-2K^b^ molecule resulted in a very drastic reduction of the Ad-YF NS3 induced protection in vivo (10% in H-2K^b^ KO mice vs. 100% in B6 mice), we wanted to confirm that NS3 vaccinated H-2K^b^ deficient mice would not be able to control YF-17D infection of the CNS following intracranial challenge. Finally, to further narrow down the identity of the involved T cells, we made an adenoconstruct encoding a truncated version of NS3 (Ad-YF NS3 _(201–325)_), and the protection associated with this vector was compared to that of the full length NS3 vector. Thus, we analyzed the viral loads in the brains of wt B6 mice that had been immunized with Ad-YF NS3 or Ad-YF NS3 _(201–325)_ 14 days prior to intracranial YF challenge, as well as of H-2k^b^ KO mice immunized with Ad-NS3 prior to intracranial challenge. Unvaccinated wt B6 mice served as controls. Viral titers were analyzed 7 days post challenge as this represents the latest time point for unbiased analysis of acutely challenged mice that would invariably succumb between day 8 and 9 p.c. As expected, the unvaccinated mice uniformly displayed a high viral burden in the CNS (10^6^−10^7^ pfu/g of brain), while the infection appeared to be partly controlled in the brains of Ad-YF NS3 immunized mice with viral titers about 3 logs lower than in the unvaccinated controls (Fig 5). Interestingly, vaccination of wt B6 mice with the truncated version of NS3 (Ad-YF NS3 _(201–325))_ also resulted in a significantly reduced viral burden in the brain as compared to unvaccinated mice (Fig 5), albeit not quite to the same degree as observed in full-length vaccinated mice. Notably, NS3 immunized H-2K^b^ deficient mice all displayed high viral loads in the CNS, almost at the same level as in unvaccinated mice. The same difference in the capacity to control YFV infection was observed when wt and H-2k^b^ deficient mice had been vaccinated 60 days prior to challenge (see S1 Fig). These results confirmed that vaccination with the Ad-YF NS3 vector could induce protection through CD8^+^ T cells predominantly specific for H-2K^b^ restricted epitopes. Furthermore, the finding that mice given Ad5 encoding the truncated or the full length form of the NS3 protein displayed overlapping reductions in viral loads corroborated the hypothesis that the NS3 _(268–275)_ specific CD8^+^ T cells represent a key subset of CD8+ T cells pivotal in Ad-YF NS3-induced antiviral immunity, albeit additional epitopes may also play a minor role.

### Protection induced by Ad-YF C,M,E relies on both T and B cell responses

**Fig 6:**
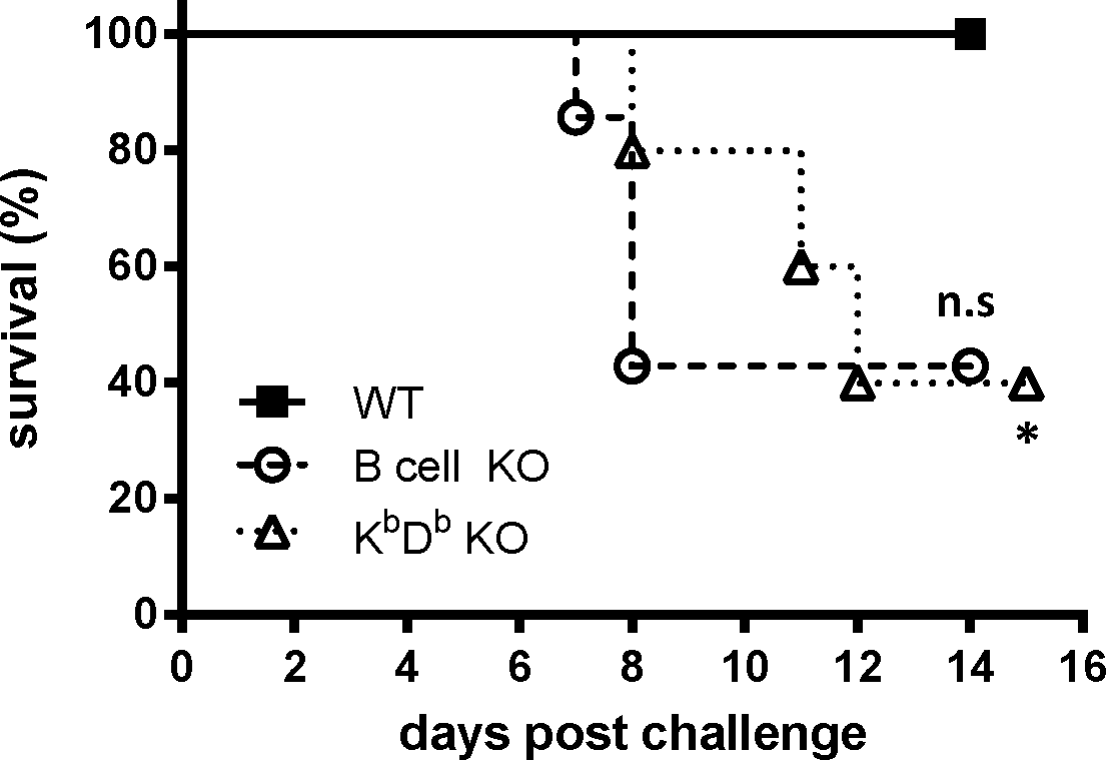
Protection from lethal disease following vaccination with Ad-YF C,M,E relies on both T- and B-cell responses. WT B6 mice, B cell KO mice and H-2K^b^/D^b^ KO mice were vaccinated with Ad-YF C,M,E and intracranial challenged 14 days later with YFV. Survival curves for each group of vaccinated KO mice are compared to that of vaccinated WT B6 mice. WT: n = 10; B cell KO: n = 7; H-2K^b^/D^b^ KO: n = 5. The depicted data are pooled from two independent experiments.

Our best Ad-YF vaccine candidate in terms of in vivo protection appeared to be the vector encoding the viral structural proteins. Given that vaccination with this construct unlike the NS3 targeted construct induced a significant humoral response, we hypothesized antibodies were mainly responsible for the protection observed in Ad-YF C,M,E vaccinated mice. To test this hypothesis, we compared the outcome of intracranial YF challenge in vaccinated B-cell KO mice, H-2K^b^/D^b^ double KO mice and wt B6 mice. Mice were injected with 2x10^7^ pfu of Ad-YF C,M,E in the f.p. two weeks prior to intracranial challenge, and the clinical outcome was monitored for 15 days after challenge. Interestingly, lack of B cells only resulted in diminished, but not abrogated protection (about 43% survival, Fig 6), relicating what we have recently observed regarding mice vaccinated with YF-17D [22]. In the case of MHC class I KO mice lacking both H-2k^b^ and H-2D^b^ molecules and with no known defects in B cell/antibody responses, we observed that protection was significantly reduced (40% survival) as compared to survival of all immunized wt B6 mice (Fig 6). Taken together these data suggested that a combination of CD8^+^ T-cell and B-cell responses are required for protection as induced by Ad-YF C,M,E, thus completely mimicking the protective response in YF-17D vaccinated animals [22].

### Protection in Ad-YF C,M,E vaccinated mice reflects a combination of CD8 T-cell mediated and humoral immunity, while protection following NS3 entirely relies on CD8^+^ T cells

**Fig 7:**
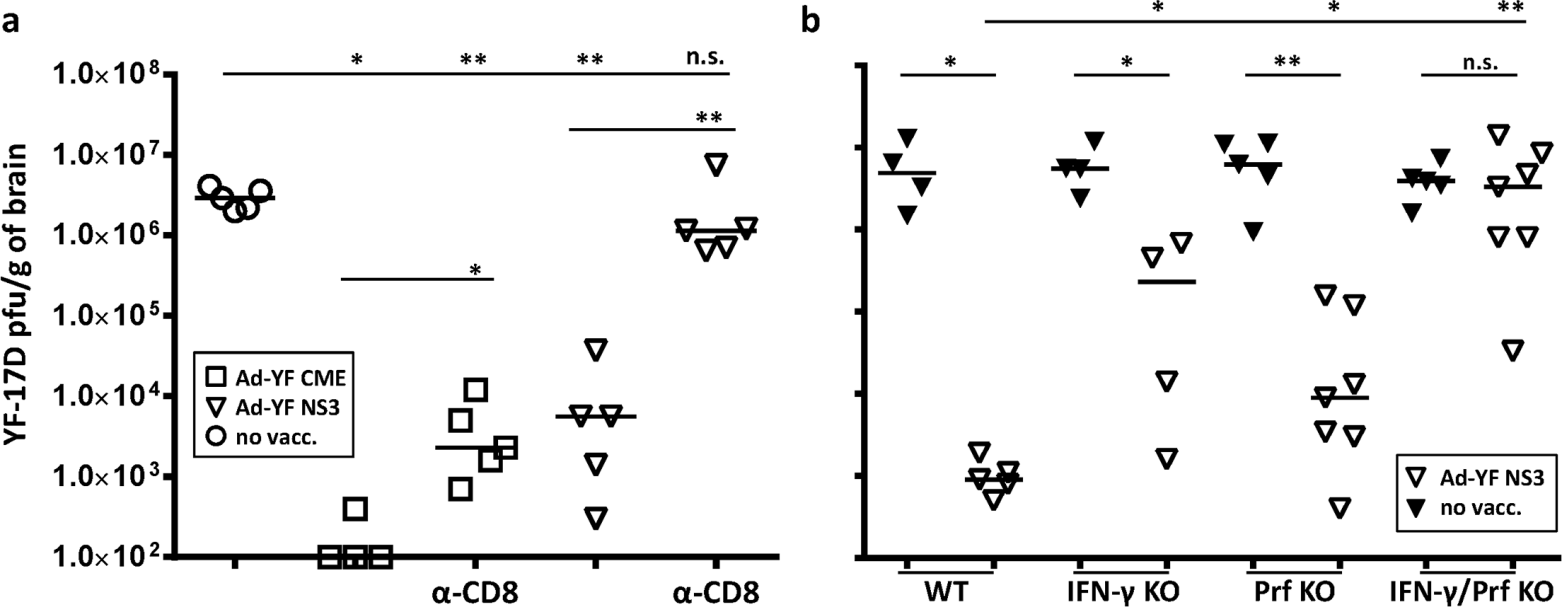
Protection in Ad-YF C,M,E vaccinated mice reflects a combination of CD8 T-cell mediated and humoral immunity, while protection following NS3 entirely relies on cytolytic and/or cytokine producing CD8^+^ T cells. **a**) WT B6 mice were vaccinated with Ad-YF C,M,E or Ad-YF NS3 and intracranial challenged with YFV 60 days later; part of the vaccinated animals were in vivo depleted of CD8^+^ T cells (α-CD8) 1 day prior to intracranial challenge, and again at days +1 and +4 after challenge. Viral titers in the CNS of Ad-YF vaccinated and Ad-YF vaccinated/CD8 depleted mice 7 days after challenge are compared to those in unvaccinated control animals. Dots represent individual animals. **b**) IFN-γ KO, Prf KO, IFN-γ/Prf double KO and WT B6 mice were vaccinated in the f.p. with Ad-YF NS3 and, 14 days later, intracranial challenged with YF-17D virus; viral titers in the CNS of vaccinated mice are compared to those of matched unvaccinated control animals at day 7 post YF challenge. Dots represent individual animals.

Based on the above results it would appear that both humoral and T-cell mediated immunity were involved in protecting Ad-YF C,M,E vaccinated mice, while only CD8^+^ T cells contributed significantly in Ad-YF NS3 vaccinated animals. However, as the above result were obtained using KO mice and therefore could have been biased from events taking place during the priming phase, we decided to reassert our interpretation by performing acute depletion of CD8^+^ T cells prior to intracranial challenge of both Ad-YF C,M,E and Ad-YF NS3 vaccinated wt mice. To this end, animals were vaccinated as previously described and intracranial challenged with YFV 60 days later; unvaccinated wt mice were included as controls. Part of the vaccinated mice were depleted of CD8^+^ T cells in connection to the challenge and on day 7 p.c., brains from all mice were collected and viral titers were determined. As expected (Fig 7A), unvaccinated animals all exhibited high viral loads, while most Ad-YF C,M,E vaccinated mice had completely cleared infectious virus from the CNS by day 7 p.c. In these mice CD8^+^ T-cell depletion lead to a significantly increased viral load, but brain virus titers were still several logs lower in these animals than in the unvaccinated controls; this matches what we recently observed in YF-17D vaccinated mice [22]. In contrast, but still as expected, infectious virus was detected in the CNS of Ad-YF NS3 vaccinated, CD8^+^ T-cell replete animals, but with viral loads significantly lower than in unvaccinated controls, and antiviral protection was completely abrogated following CD8^+^ T-cell depletion (Fig 7A).

To define the precise effector mechanisms elaborated by NS3-specific CD8^+^ T cells, we next analysed the virus titers in brains of Ad-YF NS3 vaccinated IFN-γ KO, Prf KO, IFN-γ/Prf double KO as well as wild type mice that were intracranial challenged 14 days post vaccination; strain-matched unvaccinated animals served as controls (Fig 7B). We observed that vaccination with Ad-YF NS3 still induced a significant, but markedly reduced protection in the absence of IFN-γor Prf. On the other hand, vaccinated double KO mice could not control YFV infection of the CNS and displayed viral loads similar to those of strain matched unvaccinated control animals (Fig 7B) confirming our earlier finding regarding survival of vaccinated double KO mice (Fig 4A).

### A single vaccination with Ad-YF vectors elicit long-lasting protection

**Fig 8:**
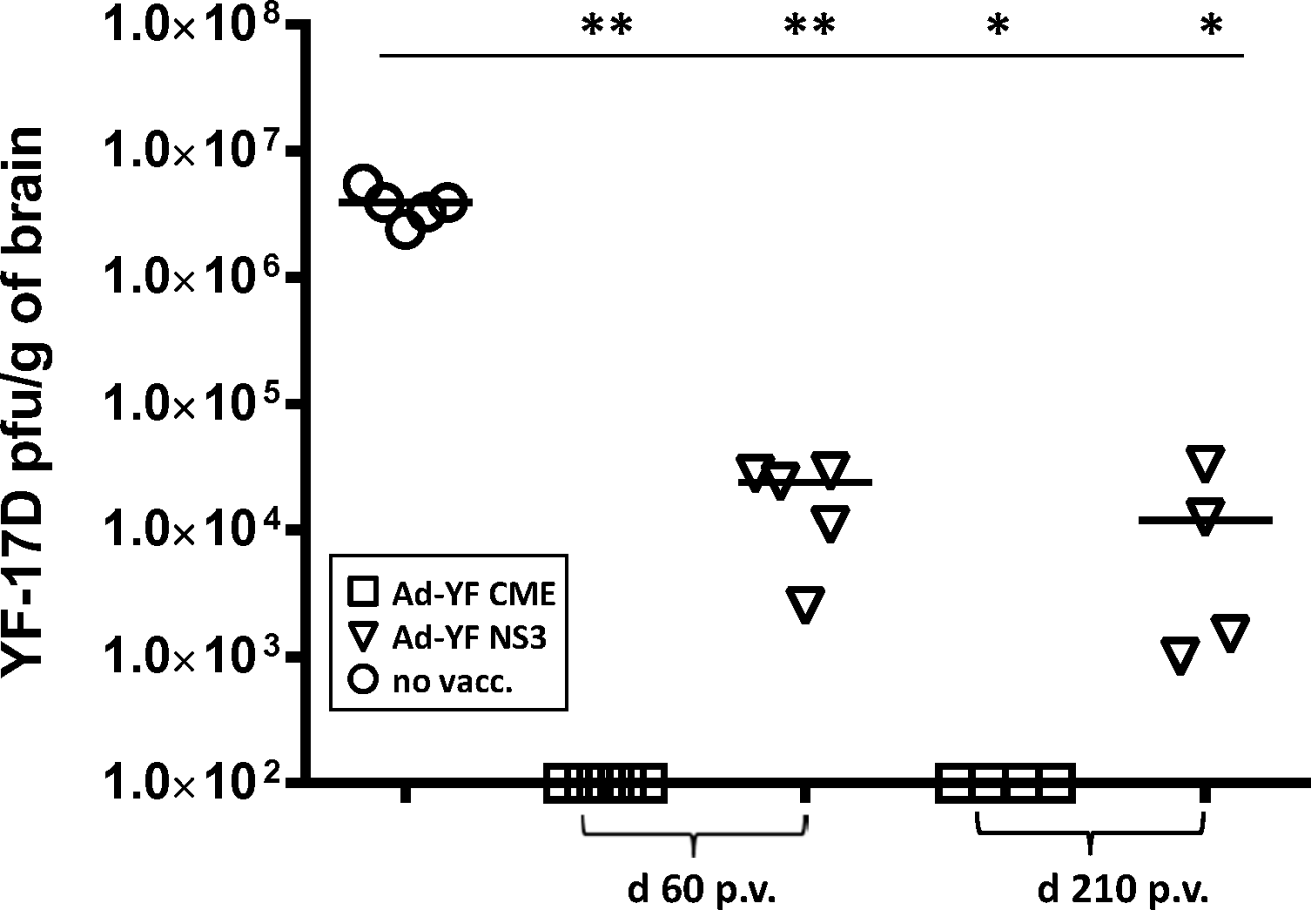
Single vaccination with either adenovector induces long-lasting protection in WT mice. WT B6 mice were vaccinated with Ad-YF C,M,E or Ad-YF NS3 and intracranial challenged with YFV 60 or 210 days later. On day 7 post challenge viral loads in the CNS of vaccinated animals are compared to those in unvaccinated control mice. Dots represent individual animals.

Finally we asked how long Ad vector-induced protection would last. To this end mice were vaccinated with each of the two adenoviral vectors as before, and 60 and 210 days later, the mice were intracranial challenged with YFV; 7 days later viral loads in the CNS of vaccinated animals were compared to those in unvaccinated controls. As can be seen in Fig 8, markedly reduced virus titers were found in both groups of vaccinated mice compared to unvaccinated controls 60 days post vaccination, and, notably, we did not observe any tendency towards a decline in vaccine-induced protection in the mice vaccinated 210 days earlier, indicating that even when acting on their own, CD8^+^ T cells have the capacity to provide long-term clinical protection in this murine model of YFV infection.

## Discussion

The first generation of viral vaccines comprised empirically attenuated live viruses, which have been documented to be very effective in preventing many infections. The yellow fever vaccine (YF-17D) belongs to this category, and with more than 500 million doses administered worldwide in the past 70 years, it stands as a paradigm for an effective vaccine [1,5]. For a number of years there was limited interest in further development of YF vaccines due to the successful application of the live attenuated vaccine. In the last decade, however, the use of the YF-17D virus has come into focus again due to the risk of serious adverse events following vaccination, and due to restrictions in immunizing certain categories, such as infants, pregnant women, elderly and immune-compromised people [31]. This has created a demand for alternative vaccine candidates [31], but only a limited number of studies have so far come up with potential new YF vaccines, and they have mostly been focused around making an inactivated vaccine inducing humoral immunity [32–34]. However, if as suggested by both of our studies [22], elicitation of an antiviral CD8 T-cell response is pivotal for full protection against YFV infection, an inactivated vaccine would not represent the best choice, whereas an Ad-vector based vaccine would seem an ideal solution. In a very recent study [11], a naked DNA vaccine encoding the E protein was tested in a murine model similar to ours and found to protect against a fatal outcome of intracranial infection; antibody levels were sufficient to explain the prevention of any mortality, whereas numbers of virus-specific T cells were quite low compared to what we obtain. Importantly, any functional importance of the induced T-cell response was not documented.

In this report, we have evaluated two prototypic Ad-based vectors encoding different YF-17D proteins. The choice of these proteins was based on our previous analysis of how the existing YFV vaccine works [22] and thus represents a translational approach to use the information previously gathered directly in the early design of a safer, but still highly efficient vaccine candidate. Our results clearly demonstrate that induction of YFV-specific CD8+ T-cell responses can lead to effective antiviral immunity in an infection model where protection until recently was ascribed entirely to neutralizing antibodies [4]. We demonstrate that a single vaccination with Ad-YF NS3, containing a conserved nonstructural viral protein, can induce almost complete protection from subsequent intracranial YFV challenge in wt B6 mice, and that CD8^+^ T cells specific for H-2K^b^-restricted viral epitopes are the major effectors in the vaccine-induced immunity. These findings confirm and extend recent results obtained in similarly challenged YF-17D vaccinated mice [22]. Here we obtained strong evidence for a primary role of antibodies in the protection against YF disease in mice, but also found evidence pointing to a supporting role for virus-specific CD8^+^ T cells. Thus, an accelerated influx of virus-specific CD8^+^ T cells into the infected CNS was revealed in vaccinated mice, and CD8^+^ T-cell depletion significantly reduced antiviral protection [22]. In the present study we observed that CD8^+^ T-cell depletion completely abolished virus control in Ad-YF-NS3 vaccinated mice, and under these conditions we could show that absence of either IFN-γ or perforin significantly impaired virus control, whereas deficiency of both completely prevented a reduction in CNS viral load at 7 days post challenge. This is in slight contrast to our previous findings in YF-17D vaccinated mice in which case only mice lacking both IFN-γ and perforin were found to be significantly impaired in their ability to control infection of the CNS [22]. This apparent discrepancy probably reflects that YD-17D vaccinated mice unlike NS3 vaccinated mice have preexisting neutralizing antibodies, which also contribute to the observed virus control and hence lead to a blurring of the results [22]. Thus taken together our data convincingly demonstrate that T-cell released effector molecules together with antibodies represent an integrated component of the adaptive immune response to YFV in CNS.

To further address whether this focused T-cell response could be responsible for the in vivo protection conferred by Ad-YF NS3 vaccination, we designed a vector encoding a truncated version of the protein (Ad-YF-NS3 _(201–325)_). In preliminary experiments we observed that the truncated form of NS3 induced similar numbers of NS3 _(268–275)_ specific T cells as did the full length protein (average of 5 mice/group: 10^7^ tetramer^+^ CD8 T cells/spleen in Ad-YF-NS3 _(201–325)_ vaccinated mice vs. 9x10^6^ CD8 T cells/ spleen in Ad-YF NS3 vaccinated mice). However, the immune mediated virus control in the CNS was slightly reduced in mice vaccinated with the former construct, suggesting that while NS3 _(268–275)_ specific T cells clearly represent the functionally most important subset of CD8+ T cells in NS3 immunized mice, there are likely to be additional minor specificities involved following immunization with the full length construct.

Notably, our attempt of generating a vector encoding the three YF viral structural proteins C, M and E resulted in an even more promising vaccine candidate. This was, to some extent, expected, as neutralizing Abs directed against the E protein have long been associated with protection from yellow fever [4]. However, upon immunization with Ad-YF C,M,E, we also noted a very strong CD8^+^ T cell response toward the subdominant viral epitope contained in the E protein, as compared to vaccination with YF-17D virus. CD8 T-cell depletion of these mice confirmed that antiviral protection reflected a combination of a humoral and a cell-mediated immune response similar to what we have recently found to be the case in YFV vaccinated mice [22]. Analysis of the neutralizing titers in Ad-YF C,M,E vaccinated mice revealed that such antibodies were induced, albeit not quite to the same level as found in YFV vaccinated mice.

In conclusion, our current results provide new independent support for our recent claim [22] that CD8^+^ T cells may play a significant role as effector cells against YFV infection. Still, the mechanisms of protection against intracranial challenge in mice may differ from those controlling the liver infection of humans. T-cell mediated immunity may be particularly important for controlling CNS infection owing to the blood/brain barrier, which initially limits antibody access. Most importantly, however, our findings demonstrate that by using the information previously gathered in studies of YFV vaccinated mice [22], it was possible to make promising vaccines candidates based on replication deficient adenoviral vectors, which might work—either alone (Ad-YF C, M, E) or combined (to broadened the T-cell response)—as potentially safe alternatives to the use of a live attenuated vaccine for induction of protection against YFV-induced disease; certainly, SAEs which represent visceral and neural dissemination of the attenuated YFV vaccine should be avoided. Notably, when it comes to human vaccinations there may be a need to substitute Ad5 with another adenovector due a high prevalence of antibodies against this serotype in certain human populations [35–37]. However, adenoviral vectors of non-human origin have already reached clinical testing (Ebola [38]) and several appear to match Ad5 in immunogenecity and safety [19,39]. Another critical issue may be the longevity of adenovector-induced protection in humans, e.g. adenoinduced protection towards Ebola infection is short-lived in non-human primates [18]. This should not represent a major problem, however, since numerous studies have demonstrated that adenovector priming works well in conjunction with heterologous boosting, e.g. using modified vaccinia Ankara [18]. In summary, our findings are very promising, and we believe that an adenovector based approach to develop a safer alternative for YFV vaccination deserves further evaluation in relevant animal models.

## Supporting Information

S1 Fig WT B6 mice and H-2Kb KO mice were vaccinated with Ad-YF NS3 and intracranial challenged with YFV 60 days later. Virus titers in the CNS of vaccinated animals are compared to those in unvaccinated WT B6 mice. Dots represent individual animals.
(TIFF)
