# Supplementary figures and images for: Vaccination with Replication Deficient Adenovectors Encoding YF-17D Antigens Induces Long-Lasting Protection from Severe Yellow Fever Virus Infection in Mice
Source: PLoS Negl Trop Dis. 2016 Feb 17;10(2):e0004464. doi: 10.1371/journal.pntd.0004464 (PMC4757529; doi:10.1371/journal.pntd.0004464)

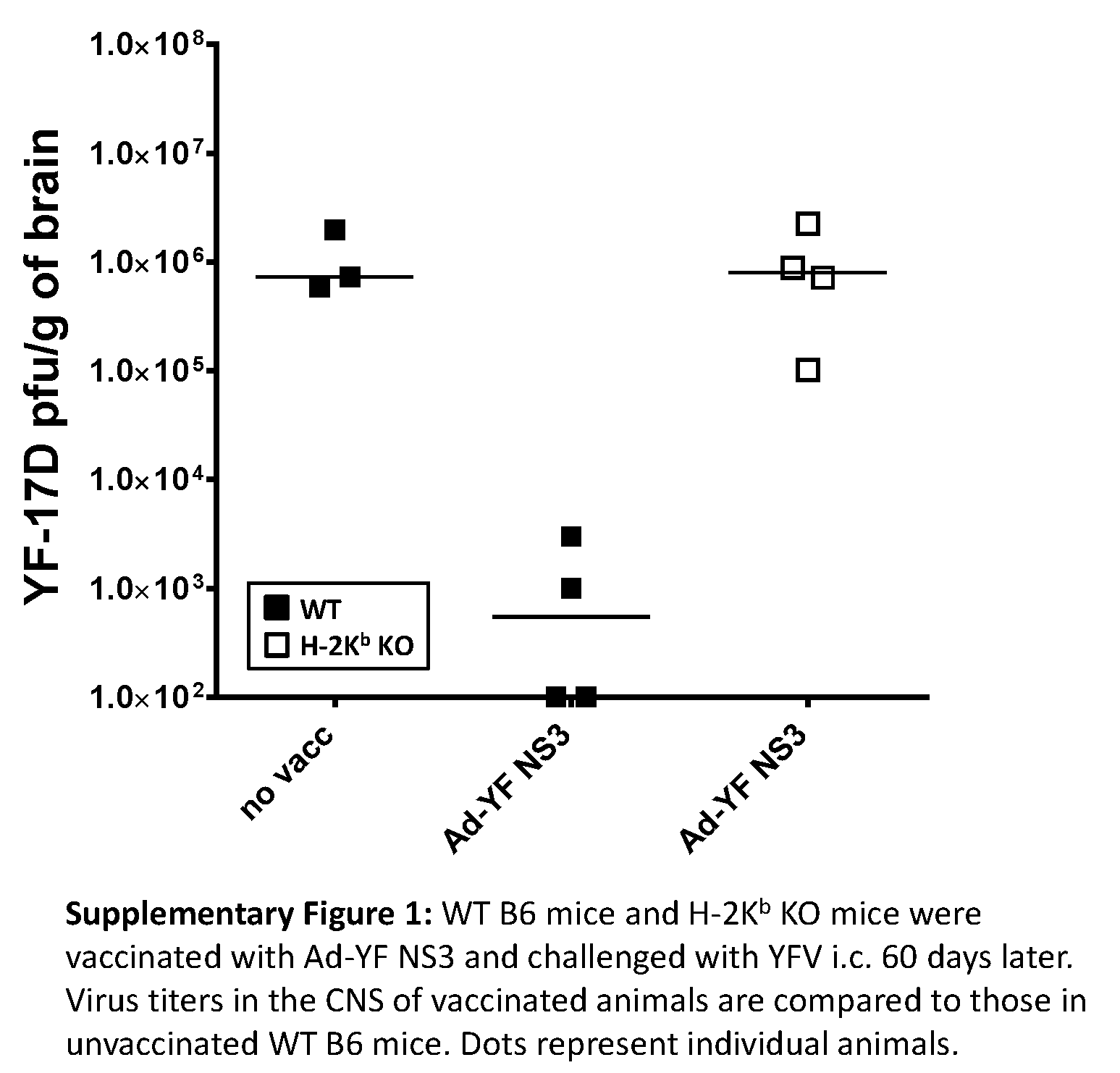

Supplement: S1 Fig — Virus titers in the CNS of vaccinated animals are compared to those in unvaccinated WT B6 mice. Dots represent individual animals. (TIFF) [file pntd.0004464.s001.tiff]
